# The Great Northern Central Hospital

**Published:** 1909-12-04

**Authors:** 


### THE GREAT NORTHERN CENTRAL HOSPITAL.

### A MODERN HOSPITAL DINNER.

Like most other matters, the hospital dinner has shown a tendency of late to fall out of favour. The cause is partly owing, no doubt, to a change in the habits of the classes from which the guests were formerly drawn. This change, coupled with the deeper interest which is now taken in hospital questions by the public generally, has created a feeling that a considerable portion of the contributions nominally raised by the dinner might be obtained, without the expense and trouble of giving such an entertainment, by making each annual meeting the occasion for a special effort. If the same enthusiasm and energy are put into the organisation of a large attendance at the annual meeting, and the presence of interesting speakers is secured, the results to a voluntary hospital have proved to be more than satisfactory where a trial has been given to this plan. The real cause, however, of the passing of the hospital dinner has been, no doubt, the absence of sufficient enterprise on the part of those responsible for holding it. This view finds justification in the circumstance that, whenever a hospital dinner has been organised under the active control of a public man of eminence who consents to take the chair, there the results have equalled, if they have not surpassed, those of the past. It is too little apprehended that nothing can be accomplished well without a great deal of hard work and personal effort. This principle applies with telling force to benefit performances or dinners or entertainments on behalf of a great hospital.

The Great Northern Central Hospital has always been fortunate in having an active committee of management, the members of which have taken the deepest personal interest in its work and progress. Last week the committee gave a dinner at the Whitehall Rooms, at which there was a record attendance, whilst the proceedings excited unusual interest. The hospital was fortunate in securing the Right Hon. Sir Edgar Speyer, Bart., as chairman. Sir Edgar Speyer made an interesting and elaborate speech, dealing with many questions of interest to hospital people, which will be found reported on another page. The Great Northern Central Hospital is unique in the circumstances that it was moved from the Caledonian Road to its present site in the crowded district of Islington with 350,000 inhabitants as the direct outcome of an agitation to bring the voluntary hospitals of London nearer to the dwellings of the people who use them most; that the buildings were entirely new; that they had been specially planned to overcome the difficulties of the site; that they consisted of rectangular and circular wards, and that those wards contained identical air, wall and superficial space per bed. It was maintained twenty years ago that the population of the district this hospital serves would certainly support and maintain it adequately. Unfortunately a great change has taken place in the character of the residents in many portions of the district, with the result that well-to-do people are far too few, and that although the hospital flourished pecuniarily for a decade or more it is now feeling the necessity for a larger revenue. The total deficit at the end of the year 1909 is estimated to amount to £14,500 in round numbers.

This was the position which Sir Edgar Speyer had to face when he consented to take the chair at the annual dinner. He threw himself heartily into the work of raising a substantial sum to liquidate the debt, devoted a great deal of time to the thorough inspection of the hospital and all its departments, and to visits which he paid to institutions in the district surrounding the hospital. We mention these facts because they show that it is still possible to obtain wealthy men, occupying a high position in the City of London, who will give both time and money in support of a voluntary hospital, and that where such support is absent, and financial misfortune follows, so far as the London hospitals are concerned it is fair to attribute it in some measure to the want of business method of the committee for the time being.

It will be seen by those who read Sir Edgar Speyer's speech that he not only gave time and thought and money, but that he brought to bear his practical business experience and made this dinner of special interest by preparing and delivering a speech which must interest a far wider audience than those present at the dinner. The effect of the speech was immediately shown by the remarks which were made by the Mayor of Islington and by the reply of the chairman of the hospital, Sir John Dickson-Poynder, M.P. These speeches were full of interest in that they debated the questions raised by Sir Edgar Speyer, so that the occasion was made of considerable practical interest and value not only to the supporters of the Great Northern Central Hospital, but to hospitals generally. We advise the managers of hospitals, who may have to organise a dinner of this kind in future, to follow the example set by Sir Edgar Speyer by reducing the toast list to the smallest possible proportions and securing two or three good speakers to deal with important points affecting the wise administration and financial stability of our hospitals. By announcing beforehand the bill of fare, they would find in all probability that the demand for tickets would be larger than they could supply, and the results in actual cash and generally cannot fail to be improved.

Sir Edgar Speyer dwelt, it will be seen, upon the essential importance for a hospital to keep out of debt. Chronic deficits are demoralising, and the duty of every committee is to keep its hospital solvent by preventing extravagance, refusing to sanction expenditure upon buildings until revenue is forthcoming to maintain them when they are erected, keeping a tight hand over the expenditure in each department, and securing the co-operation of the honorary medical and resident staff with this end in view. The contributions announced at the Great Northern Central Hospital dinner amounted to £6,471 2s. 4d. They were obtained by a number of zealous workers, as will be seen by the list, of whom the chairman was easily first. This sum, large as it was in certain aspects, was quite inadequate to wipe out a debt of £14,500. Immediately after the dinner Sir Edgar Speyer had a brief conference, which resulted in his taking steps whereby he has succeeded in raising the amount to £8,000, thus constituting a record for hospital dinners in 1909. Sir J. Dickson-Poynder, with a few friends, is working hard to raise £2,000 more, and if he succeeds in doing this, or most of it, by December 31 next, we have reason to believe that the Great Northern Central Hospital will be enabled to commence the year 1910 free from debt.

Sir Edgar Speyer has done an immense service to this hospital by presiding at its dinner. He has set an example, which we venture to hope may be followed, in commemorating the appearance of his name in the Honours List, by doing this splendid personal service in the cause of the voluntary hospitals of London. We congratulate Sir Edgar Speyer, who deserves the warm recognition of all the friends of the hospitals throughout the metropolis and the country generally. Indeed, we believe he has initiated a new and better form of hospital dinner which is likely to be generally adopted with most fruitful results for hospital revenues.

